# Population and genetic outcomes 20 years after reintroducing bobcats (*Lynx rufus*) to Cumberland Island, Georgia USA

**DOI:** 10.1002/ece3.1750

**Published:** 2015-10-12

**Authors:** Duane Diefenbach, Leslie Hansen, Justin Bohling, Cassandra Miller‐Butterworth

**Affiliations:** ^1^ U.S. Geological SurveyPennsylvania Cooperative Fish and Wildlife Research UnitThe Pennsylvania State UniversityUniversity ParkPennsylvania16802; ^2^ Los Alamos National LaboratoryMS M887P.O. Box 1663Los AlamosNew Mexico87545; ^3^ Department of Ecosystem Science and ManagementThe Pennsylvania State UniversityUniversity ParkPennsylvania16802; ^4^ Penn State Beaver100 University DriveMonacaPennsylvania15061

**Keywords:** Fecal DNA, felid, microsatellites, population genetics, population viability, reintroduction, scat, spatially explicit capture–recapture population estimation

## Abstract

In 1988–1989, 32 bobcats *Lynx rufus* were reintroduced to Cumberland Island (CUIS), Georgia, USA, from which they had previously been extirpated. They were monitored intensively for 3 years immediately post‐reintroduction, but no estimation of the size or genetic diversity of the population had been conducted in over 20 years since reintroduction. We returned to CUIS in 2012 to estimate abundance and effective population size of the present‐day population, as well as to quantify genetic diversity and inbreeding. We amplified 12 nuclear microsatellite loci from DNA isolated from scats to establish genetic profiles to identify individuals. We used spatially explicit capture–recapture population estimation to estimate abundance. From nine unique genetic profiles, we estimate a population size of 14.4 (SE = 3.052) bobcats, with an effective population size (*N*_e_) of 5–8 breeding individuals. This is consistent with predictions of a population viability analysis conducted at the time of reintroduction, which estimated the population would average 12–13 bobcats after 10 years. We identified several pairs of related bobcats (parent‐offspring and full siblings), but ~75% of the pairwise comparisons were typical of unrelated individuals, and only one individual appeared inbred. Despite the small population size and other indications that it has likely experienced a genetic bottleneck, levels of genetic diversity in the CUIS bobcat population remain high compared to other mammalian carnivores. The reintroduction of bobcats to CUIS provides an opportunity to study changes in genetic diversity in an insular population without risk to this common species. Opportunities for natural immigration to the island are limited; therefore, continued monitoring and supplemental bobcat reintroductions could be used to evaluate the effect of different management strategies to maintain genetic diversity and population viability. The successful reintroduction and maintenance of a bobcat population on CUIS illustrates the suitability of translocation as a management tool for re‐establishing felid populations.

## Introduction

**Figure 1:**
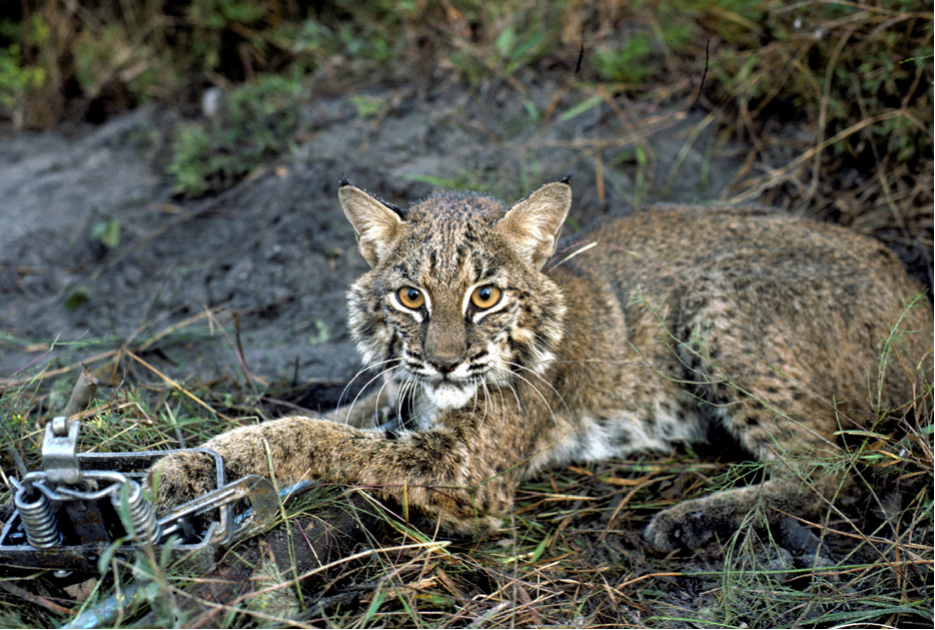
A bobcat *Lynx rufus* captured in mainland Georgia, USA, and released onto Cumberland Island, GA (1988–1989).

Bobcats *Lynx rufus* Schreber 1777 are currently the most broadly distributed native felid in North America, with a continuous distribution extending from Canada to Mexico (Larivière and Walton 1997). Historically their range included Cumberland Island (CUIS), a barrier island located along the coast of Georgia (GA), but they were extirpated by the early 20th century (Diefenbach et al. 1993). Following the loss of terrestrial predators on CUIS, white‐tailed deer *Odocoileus virginianus* and feral hogs *Sus scrofa* were implicated in the reduction of native vegetation, particularly lack of regeneration of live oak *Quercus virginiana* (Diefenbach et al. 2013). In 1988–1989, the U.S. National Park Service, which manages the island, funded the release of 32 bobcats (Fig. 1) to CUIS to restore a native predator to the ecosystem (Diefenbach et al. 1993).

In the short term, the success of a reintroduction program can be assessed by whether the first wild‐born generation breeds successfully and natural recruitment of a 3‐year breeding population exceeds mortality (Hayward and Somers 2009). To evaluate the initial success of this reintroduction program, survival, movement, and reproduction of bobcats on CUIS were monitored for 3 years immediately postreintroduction (1989–1991). Adult annual survival was high (93%), physiological condition of recaptured bobcats was excellent, and reproduction was documented (Diefenbach 1992; Diefenbach et al. 1993).

Understanding the fate of this population in response to demographic and genetic stochasticity has important implications for conservation management decisions. On a global level, 30 of the 37 species of Felidae have declining populations, and 18 are at risk for extinction in the wild (IUCN 2015). Opportunities for immigration or emigration are limited in isolated or insular populations, such as CUIS, and with habitat loss and fragmentation many endangered midsized felid species now face similar physical limitations and restricted opportunities for successful dispersal (e.g., Iberian lynx *Lynx pardinus*, Ferreras 2001). In particular, isolated populations within a metapopulation may require human intervention via translocations to maintain numeric abundance of animals (Lubow 1996) or genetic diversity (Hedrick and Fredrickson 2010). Consequently, there is a need to understand the dynamics of small populations founded by translocations to assess their viability and need for future human intervention.

Following the CUIS bobcat reintroduction, Diefenbach (1992) modeled the long‐term viability of the population. A population viability analysis (PVA) predicted a 0.32 probability of persisting 100 years, a 0.73 probability of persisting >50 years, and a median time to extinction of 65 years. This PVA predicted the abundance of bobcats would decline during the first 10 years and then average at about 12–13 bobcats, with fluctuations ranging from as many as 27 bobcats (1 bobcat/2.6 km^2^) to multiple bottlenecks of fewer than five bobcats. Such small population size could facilitate inbreeding depression and genetic drift that would further reduce the viability of this population.

Although post‐reintroduction research on the CUIS population of bobcats examined behavioral and ecological processes such as social organization (Diefenbach et al. 2006), changes in prey choice and abundance (Baker et al. 2001; Diefenbach et al. 2006), and trophic cascades (Nelms 1999; Diefenbach et al. 2009), no estimation of bobcat population size or genetic diversity has been conducted in more than 20 years since reintroduction. The purpose of this study was to collect fecal material found on the island and to use a combination of molecular and modeling techniques to (1) estimate the abundance; and (2) document levels of genetic diversity of bobcats currently on CUIS. The bobcat reintroduction to CUIS provided an opportunity to assess the outcome of translocation as a management tool for re‐establishing felid populations and assess the outcomes of a reintroduction program that has received no additional intervention since the initial releases. Such information could be used to help guide future management decisions of this specific population and to inform future felid translocation efforts.

## Materials and Methods

### Study area

Cumberland Island is a coastal barrier island, 25 km long, located 0.5 km north of the Georgia–Florida border. Its eastern shores face the Atlantic Ocean, and to the west, it is separated from mainland Georgia by 2–4 km of salt marsh and open water. Little Cumberland Island lies to the north, separated from the main island by 0.25 km of salt marsh and a tidal creek. The combined area of upland habitats on both islands is approximately 6935 ha. The climate is warm temperate to subtropical. Both islands contain a variety of habitats, including sandy beach and interdune meadow, interior maritime forests (dominated by live oak and pine *Pinus* sp.), scrub‐shrub thickets, freshwater wetlands, and salt marsh.

### Bobcat reintroduction

In September 1988, a scent survey was conducted on CUIS to verify that no terrestrial predators were present on the island prior to reintroducing bobcats (Diefenbach et al. 1994). Personnel also searched for bobcat sign (tracks and scat) during regular fieldwork on the island from July to August 1988, and none was identified (Diefenbach et al. 1994). The reintroduction of bobcats to CUIS has been described in detail previously (Diefenbach et al. 1993). Briefly, bobcats were captured (Fig. 1) in the coastal plain of mainland Georgia, which has similar habitat and climate to CUIS, and each animal was fitted with a radio collar. Because the bobcats were trapped in several geographically dispersed areas, there was likely a good admixture of genetic diversity in the released individuals. Between October and December of 1988 and 1989, the bobcats were released onto the island at approximately 30‐day intervals in groups of four to six animals. All bobcats were transported to the island and released immediately. In total, 32 adult (>1 year old) bobcats (15 males and 17 females) were released on CUIS. During 1989–1991, five bobcats (three males and two females) died and one female returned to the mainland.

### Sample collection

**Figure 2:**
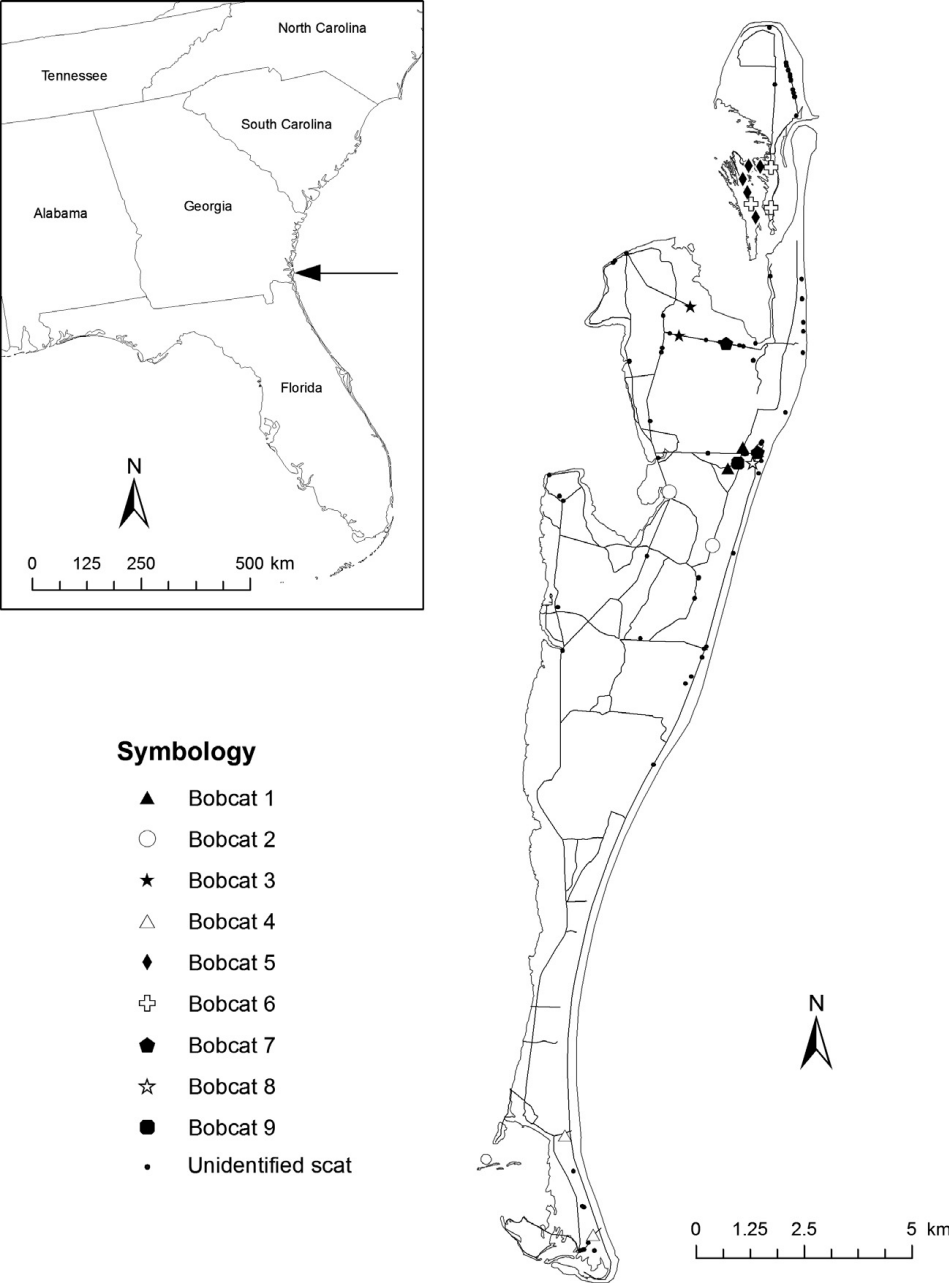
Map of Cumberland Island, GA, USA, showing roads and trails searched for scats and the locations of all scats that were collected (2011–2012). Twenty‐one of these yielded usable DNA from which nine bobcat individuals were identified. The remainder were degraded and therefore were unidentified.

We returned to CUIS from 29 December 2011 to 2 January 2012 to collect scat samples from the current population of bobcats, 23 years post‐reintroduction. We established transects (Fig. 2) along roads, hiking trails, and the interdune meadow, which were traversed by one person walking for 2–3 h. We searched each transect for scat at least twice. We recorded the spatial coordinates of all scats using a Garmin 12XL handheld GPS (Garmin Schaffhausen, Switzerland).

For each scat that likely contained viable DNA (i.e., was not severely degraded by environmental conditions), we scraped a sample of the exterior surface using a scalpel blade and stored it at room temperature in a vial containing silica desiccant beads. Handlers replaced latex gloves and sampling tools after each collection to prevent contamination between samples. In most instances, individual scats were readily identifiable, but where multiple scats were deposited in close proximity, we collected samples only from scats likely coming from a single deposition; otherwise, no sample was collected.

### Laboratory procedures

We extracted DNA from scats using the QiAmp Stool Kit (Qiagen, Valencia, CA) in a laboratory dedicated to low‐quality DNA samples. We included negative controls to monitor for contamination. Coyotes *Canis latrans* are present on CUIS, and both species produce scats similar in size and appearance that cannot always be distinguished morphologically. We used a mitochondrial DNA (mtDNA) control region fragment analysis test for species identification (Onorato et al. 2006). The primers used in this test amplify a portion of the control region that is conserved across many North American mammalian carnivores and produces polymerase chain reaction (PCR) products of distinct sizes for different species. Fragments produced by bobcats and coyotes differ by 16 base pairs (bp) in size, which allowed us to discriminate scats between species.

Once we identified scats to the species‐level, we amplified DNA from each bobcat scat at a suite of 14 microsatellite loci described by Reding et al. (2012). These loci were amplified in seven multiplexes containing varying numbers of loci (See Table S1 in Supporting Information) using the Qiagen Multiplex Kit (Qiagen). Amplification conditions are described in Supporting Info.

We initially screened bobcat scats at two separate PCR multiplexes (multiplexes 1 and 2), each of which contained primers for three loci (Table S1). Each screening multiplex was amplified in at least three independent PCRs to minimize genotyping errors. Many scats failed to amplify at any locus and were subsequently excluded. Only scats that amplified at four or more loci were included in further analyses. After this initial screen, we amplified successful bobcat scats at an additional eight loci, within five multiplexes (Table S1). Once again, each locus was amplified a minimum of three times in independent PCRs.

### Population genetics

We calculated amplification and genotyping rates on a per locus basis using data for multiplexes 1 and 2, because those were the only loci amplified for all scats. The remaining multiplexes were selectively applied to samples that were successfully amplified at the first two multiplexes; thus, their rates are likely positively biased.

We accepted a genotype at an individual locus only if that genotype was observed in three independent PCRs. For putative homozygous genotypes, a single allele had to be observed in all PCRs in order to be accepted as homozygous. Once genotypes were identified at each locus, we generated a consensus multilocus genotype for every scat. Only scats that produced consensus genotypes at six different loci were included in further analyses, which minimized the probability (*P *< 10^−6^) of identifying siblings as the same individual.

We used GENALEX (Peakall and Smouse 2006) to perform a matching analysis with these six locus genotypes to identify duplicate genotypes. To be grouped together as a single consensus genotype, scat genotypes had to match at a minimum of five loci. With these duplicate genotypes, we then generated composite genotypes representing unique animals in the population. We also used GENALEX to estimate deviation from Hardy–Weinberg equilibrium and measures of heterozygosity and allelic richness (the total number of alleles in the population) for each locus.

We estimated pairwise relatedness (*r*) and inbreeding coefficients because we expected many of the individuals in this isolated, restricted population to be related. For relatedness we used two methods: the maximum‐likelihood method employed by ML‐Relate (Kalinowski et al. 2006) and the triadic likelihood estimator employed by COANCESTRY (Wang 2007, 2011). We used the triadic likelihood method to estimate inbreeding coefficients (*F*) based on allele frequencies. The inbreeding coefficient, *F*, is the probability that an individual is homozygous (i.e., has two identical alleles) at any particular gene locus because its parents were related, that is, the alleles are identical by descent from a common ancestor. In these analyses, we utilized true allele frequencies with 100 simulated reference individuals and 100 bootstrapping replicates to estimate confidence intervals. We used GENALEX to estimate population level inbreeding (*F*_IS_) to measure the extent of homozygous excess relative to allele frequencies, where *F*_IS_ is the proportion of the genetic variance in a subpopulation that is contained within an individual, and high *F*_IS_ values imply inbreeding.

### Population estimation

We estimated the abundance of the present‐day bobcat population on Cumberland Island (23 years post‐reintroduction) using the spatially explicit capture–recapture population estimation program, secr (version 2.9.0, Efford et al. 2009) implemented in R (R Development Core Team 2005). We divided transects into 749 sections of 200 m each, and defined the coordinates of the midpoint of the transect section as the location of a proximity detector. The location of each scat was assigned to the nearest 200 m transect segment. Paths searched along the interdune meadow were not as well defined as along roads and differed upon each visit. Therefore, for interdune meadow transects, we plotted a single transect along the long axis of the habitat and assigned the location of each scat to the nearest transect (Fig. 2). Although this introduced error in the location of scats, it was usually less than the transect segment length (200 m) because interdune meadow habitats were linear and generally <200 m wide.

As a comparison to our mark–recapture population estimates, we estimated effective population size (*N*_e_) to provide an indication of the effective number of bobcats that have contributed to the current gene pool. We used two methods implemented in program NeEstimator 2.01 (Do et al. 2014): one based on heterozygous excess (Pudovkin et al. 1996; Zhdanova & Pudovkin 2008) and a second based on molecular co‐ancestry (Nomura 2008).

## Results

### Amplification and genotyping success

We extracted DNA from 117 scats that were collected on the island (Fig. 2). Based on the mtDNA fragment test, we determined that 45 scats were from bobcats. An additional 46 originated from coyotes. The remaining scats did not produce readable DNA fragments. For the two screening bobcat multiplexes 1 and 2, PCR amplification rate for individual loci varied between 31% and 49% (Table S1). Genotyping rates were comparable: between 37% and 42% of scats produced genotypes for each locus, except locus FCA132, which was genotyped in only 18% of the samples. Twenty‐three scats produced genotypes at five or more loci. Two loci in the additional five multiplexes, FCA740 and FCA391, did not amplify in any samples and were removed from the dataset. After amplification at the additional multiplexes, 21 scats produced genotypes at six or more loci.

### Population genetics

We identified nine unique bobcat genotypes from the 21 scats (Fig. 2) that were reliably amplified at six or more loci. On average, each genotype was observed in 2.3 different scats, although two genotypes were observed in only a single scat. Every unique genotype was amplified at a minimum of seven and a maximum of 12 loci (mean = 10.2).

None of the loci deviated from Hardy–Weinberg equilibrium (*P *≥* *0.055). The average number of alleles observed per locus (allelic richness, AR) was 3.67 (range 2–5). Combined across all loci, observed heterozygosity (*H*_O_) was 0.742 (±0.074 SE), and unbiased expected heterozygosity (*H*_E_) was 0.631 (±0.053 SE). The *F*_IS_ was −0.255 (±0.065 SE), indicating an excess of heterozygotes.

**Figure 3:**
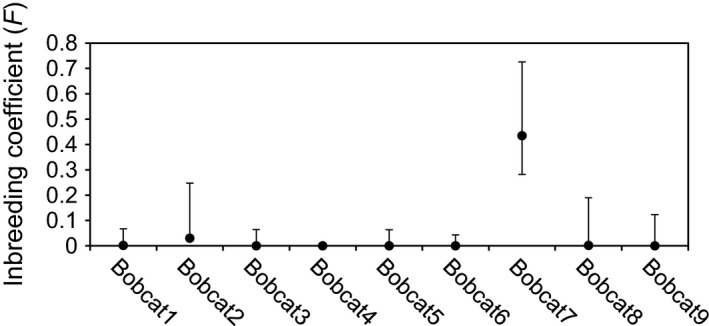
Estimates of inbreeding (*F*) with 95% confidence intervals for each bobcat from Cumberland Island, Georgia USA, 2011–2012.

Overall, we found low levels of inbreeding and obtained similar relatedness (*r*) values from ML‐Relate and COANCESTRY (Pearson's correlation coefficient between estimators was 0.785). The average value of *r* from ML‐Relate was 0.155 (SD = 0.248), and the average *r* from COANCESTRY was 0.077 (SD = 0.161). Of the 36 potential pairwise comparisons between individuals using ML‐Relate 27 (75%) produced *r*‐values close to or equal to zero, indicating the individuals were unrelated (Table 1). However, eight relationships produced *r*‐values greater than 0.5, which is indicative of parent‐offspring or full sibling relationships. The results of ML‐Relate indicated several related groups of individuals (Table 2). Individuals 1, 3, 4, and 6 formed a related group of siblings, with 1 and 6 forming a parent‐offspring pair. Additional parent‐offspring pairs were formed by individuals 2 and 5, individuals 4 and 8, and individuals 8 and 9. Using COANCESTRY, for eight of nine bobcats the individual *F*‐values (inbreeding coefficients) were <0.03 and 95% confidence intervals overlapped 0, indicating no inbreeding (Fig. 3). One individual, Bobcat 7 had an *F* estimate of 0.4343 (95% CI = 0.282–0.726) and was likely inbred.

**Table 1:** Estimates of pairwise relatedness (*r*) produced by the maximum‐likelihood method implemented in ML‐Relate (above the diagonal) and the triadic likelihood method implemented in COANCESTRY (below the diagonal) for nine bobcats from Cumberland Island, Georgia, USA, 2011–2012

	Bobcat 1	Bobcat 2	Bobcat 3	Bobcat 4	Bobcat 5	Bobcat 6	Bobcat 7	Bobcat 8	Bobcat 9
Bobcat 1	–	0	0.74	0.54	0	0.6	0	0	0
Bobcat 2	0	–	0	0	0.5	0	0	0	0
Bobcat 3	0.73	0	–	0.63	0	0.72	0	0.1	0
Bobcat 4	0.16	0.01	0.14	–	0.03	0.56	0	0.41	0
Bobcat 5	0	0.24	0	0	–	0	0	0.13	0
Bobcat 6	0.11	0.01	0.29	0.58	0	–	0	0.12	0
Bobcat 7	0.13	0	0.06	0	0	0	–	0	0
Bobcat 8	0	0	0	0.02	0.02	0	0	–	0.5
Bobcat 9	0	0	0	0	0	0	0	0.30	–

**Table 2:** Matrix of pairwise estimates of mostly likely relationships between individual bobcats produced by ML‐Relate (U = unrelated; FS = full sibling; PO = parent‐offspring), Cumberland Island, GA USA, 2011–2012

	Bobcat 1	Bobcat 2	Bobcat 3	Bobcat 4	Bobcat 5	Bobcat 6	Bobcat 7	Bobcat 8	Bobcat 9
Bobcat 1	–	U	FS	FS	U	PO	U	U	U
Bobcat 2			U	U	PO	U	U	U	U
Bobcat 3				FS	U	FS	U	U	U
Bobcat 4					U	FS	U	PO	U
Bobcat 5						U	U	U	U
Bobcat 6							U	U	U
Bobcat 7								U	U
Bobcat 8									PO

### Abundance estimate

We used the 21 scat locations from nine different bobcats (Fig. 2) to estimate abundance (Fig. 3). We estimated 14.41 bobcats (SE = 3.052, 95% CI = 1.9–24.2) on the island. We used a half‐normal detection function and estimated *σ *= 772.9 m (SE = 130.7). For effective population size (*N*_e_), the heterozygous excess method produced an estimate of 5.0 effective breeders (95% CI = 2.7–∞) with a mean weighted *D* of 0.11. The coancestry method estimated 8.4 effective breeders (95% CI = 3.8–14.7).

## Discussion

### Current abundance and effective population size

Our estimate of approximately 14 bobcats in 2012 is consistent with Diefenbach's (1992) PVA prediction that, following an initial decline over the first 10 years, the reintroduced population would average 12–13 individuals after 20 years. Although the two estimates of effective population size (*N*_e_) size varied, the ratio of effective population size to total or census population size (*N*_e_/*N*_c_ = 0.36–0.60) is relatively high for a vertebrate, which is typically *N*_e_/*N*_c_ = 0.10–0.11 (Frankham 1995). However, it is similar to estimates for several endangered felid species, including ocelots (*N*_e_/*N*_c_ = 0.37, Ludlow and Sunquist 1987; Janečka et al. 2008), tigers (*N*_e_/*N*_c_ = 0.40, Smith and McDougal 1991), and Florida panthers (*N*_e_/*N*_c_ = 0.25–0.5, Seal et al. 1989). Our estimate of effective population size, along with the observed heterozygote excess, suggests there was significant variance in the reproductive success of the founders (Hedrick 2005). To our knowledge, there is no published report of effective‐to‐population size ratio for another bobcat population, so we have no direct comparison for our value.

Behavioral traits, such as reproductive suppression and avoidance of breeding with relatives, can reduce the number of individuals that contribute to the genetic composition of a population (Anthony and Blumstein 2000). The low *N*_e_ but high heterozygosity, low inbreeding, and heterozygote excess could be the result of behavioral inbreeding avoidance. Female bobcats require an established home range in order to breed (Janečka et al. 2006). During 1989–1991, Diefenbach (1992) reported an inverse density‐dependent relationship between bobcat density and reproduction. Also, Diefenbach et al. (2006) reported failure of bobcats on the island during 1989–1991 to maintain home ranges that excluded conspecifics of the same sex. Diefenbach et al. (2006) hypothesized that reduced reproduction under high densities was the result of a failure of breeding females to maintain home ranges exclusive of other females. The number of bobcats released likely exceeded the long‐term carrying capacity of the island (Diefenbach 1992), and therefore, the population has been operating at or beyond habitat saturation since reintroduction. Most new female recruits likely settled within the existing ranges of other bobcats, which would limit overall reproductive output and restrict the number of breeders contributing to the population.

Population trajectories of the bobcats relative to those predicted by the PVA (Diefenbach 1992) may be influenced further by the presence of coyotes on the island. Scent‐surveys and manual searches for tracks and scat conducted for 3 years after bobcat reintroduction indicated that they were initially the only terrestrial predators on CUIS (Diefenbach et al. 1994). However, coyotes immigrated to Cumberland Island sometime after 1999 and have since established a year‐round breeding population (Diefenbach et al. 2013). Declines in bobcat populations have been associated with increasing coyote populations (e.g., Linhart and Robinson 1972; Litvaitis and Harrison 1989), and coyotes have been documented killing bobcats (Knick 1990), but other studies found no evidence of negative interactions between these species (Lovell et al. 1998; Neale and Sacks 2001). Sympatric carnivores are known to partition their habitat in both space and time (e.g., cougars and jaguars; Scognamillo et al. 2003), thereby limiting co‐occurrence and competition for common prey species, and this has also been documented for bobcats and coyotes in Arizona (Reed 2011). In an island population with limited opportunities for dispersal and emigration, and potentially limited food resources, the competitive presence of coyotes on CUIS may adversely affect the bobcat population. Coyotes may reduce productivity of native prey species (Kilgo et al. 2012) and create another pathway for trophic‐level effects to occur on the island (Diefenbach et al. 2013).

### Genetic attributes of bobcat population

The CUIS bobcat population has similar levels of observed heterozygosity, *H*_O_ (0.742) but lower allelic richness, AR (3.67) compared to bobcat populations sampled in continental‐wide surveys (Croteau et al. 2012; Reding et al. 2012). Reding et al. (2012) reported an overall *H*_O_ = 0.736 and AR = 11.78 for their continental‐wide survey of 1680 bobcats examined using the same microsatellite loci as our study. The CUIS bobcats have reduced allelic diversity compared to that reported for individual populations sampled on the mainland (e.g., Janečka et al. 2007; Millions and Swanson 2007; Croteau et al. 2010; Lee et al. 2012; Reding et al. 2013), including individual populations from the southeastern U.S. (*H*_O_ = 0.736, AR = 6.49) sampled by Reding et al. (2012). The high heterozygosity, low allelic richness, and high heterozygote excess indicated by a negative *F*_IS_ value (*F*_IS_ = −0.255) suggest a genetic bottleneck has occurred on CUIS.

Despite a low effective population size, the CUIS bobcat population does not yet exhibit much genetic evidence of inbreeding. We found that the population is composed mostly of unrelated individuals with a few notable exceptions (individuals 1, 3, 4, and 6; Table 2). The average ML‐Relate relatedness (*r *=* *0.155) was higher than that previously reported for other populations of bobcats (Janečka et al. 2007). It indicated the average relationship among bobcats was equivalent to first cousins, although several individuals had ML‐Relate estimates higher than would be expected for parent‐offspring and full sibling relationships (i.e., *r *>* *0.5; Tables 1 and 2). However, the triadic estimator from COANCESTRY, which accounts for both inbreeding and genotyping errors (Wang 2007), indicated only two relationships with *r *>* *0.5 (Bobcats 1 and 3: *r *=* *0.733, Bobcats 4 and 6: *r *=* *0.576; Table 1). One individual, Bobcat 7, had an inbreeding coefficient (*F *=* *0.4343, Fig. 3) that is equivalent to more than two generations of full sibling mating (Falconer 1989).

In the long term, the viability of the CUIS bobcat population will likely be challenged by its small size. Assuming the genetic diversity of the founder population was similar to that of bobcats on the mainland, discussed above, the present‐day island population has potentially lost approximately 50% of its former allelic diversity. Because small populations are particularly susceptible to the effects of genetic drift, this loss of allelic diversity may be associated with the loss of beneficial alleles, which could significantly impact future health. The effective population size was below typical recommendations for small population persistence (Frankham et al. 2014). Although only one individual showed signs of being inbred, this represents over 10% of the detected individuals, and new individuals to the population are unlikely to arrive via natural immigration from the mainland. Although CUIS was historically occupied by bobcats, immigration and emigration rates between the mainland and island are unknown. During the initial reintroduction, one female bobcat swam from the island to the mainland soon after release (Diefenbach et al. 1993), but otherwise there has been no documented dispersal or immigration. Historically, Cumberland Island likely supported a bobcat population with reduced diversity due to its isolation, which was sustained by periodic genetic inputs from the mainland (Frankham 1997; Johnson et al. 2000), or periodically became extinct until new individuals immigrated. Given that human development on the mainland and widening of the intracoastal waterway channel have likely reduced the potential for natural colonization, we predict artificial supplementation via translocation from the mainland may be necessary to prevent inbreeding depression and to maintain long‐term viability of the population.

### Species reintroduction as an experiment

Increasing isolation and fragmentation of wildlife populations reduces genetic diversity and facilitates inbreeding, which then initiates an extinction vortex (Keller and Waller 2002; O'Grady et al. 2006). Such extinction processes have been relevant to the conservation of endangered felids (Janečka et al. 2008, 2014; Joshi et al. 2013; Ernest et al. 2014), including species such as the Iberian lynx *Lynx pardinus*, Eurasian lynx *Lynx lynx,* and Florida panther *Puma concolor coryi*, which have experienced severe population bottlenecks (Schmidt et al. 2011; Palomares et al. 2012; Casas‐Marce et al. 2013). These species had *H*_O_ prior to reintroduction or translocation programs that varied from 0.167 for the Florida panther, (Johnson et al. 2010) to 0.31 for the Iberian lynx (Palomares et al. 2012; Casas‐Marce et al. 2013). There are two remaining remnant populations of the Iberian lynx, which show evidence of inbreeding depression, including reduced genetic diversity (Palomares et al. 2012), compromised immunity (Meli et al. 2010), and poor semen quality (Ruiz‐López et al. 2012). Therefore, translocations and supplementation of wild populations with captive‐bred individuals may be necessary for long‐term conservation of this species (Casas‐Marce et al. 2013).

The Iberian lynx, Florida panther, and Eurasian lynx populations have undergone bottlenecks for a longer time period than the bobcats on CUIS, which may explain why their genetic variability remains lower than that of the CUIS bobcats. However, despite the considerably larger sizes of their bottlenecked populations relative to that of the CUIS bobcats, and that some (e.g., the Iberian lynx) exist as part of a metapopulation, they have nevertheless experienced significant loss of genetic diversity over time. It is likely, therefore, that the small, isolated island bobcat population will follow a similar trajectory. Continued monitoring of CUIS bobcats could determine when genetic diversity declines, and additional introductions of individuals may be effective in maintaining genetic diversity and increasing population viability.

Genetic rescue, in which the purposeful introduction of new individuals provides an input of genetic diversity, can improve the viability of a population (Hogg and Forbes 2006; Hedrick and Fredrickson 2010; Whiteley et al. 2015). For example, the translocation of eight Texas panthers to Florida in 1995 has led to the genetic restoration of the previously highly inbred Florida panther. After 15 years, heterozygosity increased from 0.167 in the original Florida panthers to 0.244 in the current admixed population (Johnson et al. 2010).

Fifteen different reintroductions, involving 170–175 Eurasian lynx, have been carried out since 1971, five of which appear to have been successful (Linnell et al. 2009). Microsatellite‐based estimates of genetic diversity in remnant populations (pre‐reintroduction) of Eurasian lynx are not available (Schmidt et al. 2011), but six lynx reintroduced to Dinaric Mountains population in Slovenia increased to 130 animals and current *H*_O_ was 0.47 (Sindičić et al. 2013). Estimates of *H*_O_ in larger *L. lynx* populations were 0.51 in Sweden and 0.69 in Russia (Rueness et al. 2003).

The effective use of genetic rescue as a conservation technique requires testing its long‐term efficacy and evaluating situations for when it is appropriate (Boyce et al. 2011; Weeks et al. 2011). However, any actions to conserve populations, let alone experiments, often have to be planned carefully for endangered species. In contrast, bobcat populations are secure across the species' range (Kelly et al. 2008). Thus, maintaining genetic diversity and overall viability in the CUIS population is not a species conservation priority. However, for managers of CUIS, which is a national park, maintaining the bobcat population has implications for ecosystem restoration efforts. Deer are a primary prey species (Baker et al. 2001), and evidence indicates that since their reintroduction to CUIS, bobcats have caused a trophic cascade (Diefenbach et al. 2009) by reducing deer abundance and facilitating oak regeneration (Nelms 1999). This top‐down influence of terrestrial predators has been reported in other island systems (Terborgh et al. 2001).

Translocation success is highly variable in mammals, and the impacts of a release are rarely measured across genetic and demographic scales (Wolf et al. 1998; Fischer and Lindenmayer 2000). Cumberland Island provides an opportunity to test release strategies that provide a demographic and genetic boost, especially for a population already at saturation with the available environment. Although the current population of bobcats on Cumberland Island does not yet display a genetic signature of inbreeding, demographic stochasticity, genetic diversity, and presence of coyotes could influence population viability. Furthermore, given the isolation of the island, it is likely that additional reintroductions will be required in the future to ensure long‐term viability of the population.

## Data accessibility

Microsatellite genotype data, GPS location data for each scat, locations of the search transects, shapefiles of the island boundary, and the secr code used to estimate abundance have been uploaded to the Dryad digital repository (doi: 10.5061/dryad.4429j).

## Conflict of Interest

None declared.

## Supporting information


**Table S1.** PCR multiplexes and the corresponding loci associated with each reaction.
