# Supplementary material for: Population and genetic outcomes 20 years after reintroducing bobcats (Lynx rufus) to Cumberland Island, Georgia USA
Source: Ecol Evol. 2015 Oct 12;5(21):4885–95. doi: 10.1002/ece3.1750 (PMC4662311; doi:10.1002/ece3.1750)
Supplement: Supplementary file 1 — Table S1. PCR multiplexes and the corresponding loci associated with each reaction. [file ECE3-5-4885-s001.docx]

**Online Supplementary Information for Diefenbach et al.**

Amplification methods

DNA was extracted from bobcat scats collected on Cumberland Island, GA USA in 2011-2012, as described in the main text. We initially screened bobcat scats at two separate PCR multiplexes (multiplexes 1 and 2), each of which contained primers (Reding et al. 2012) for three loci (Table S1). After this initial screen, we amplified successful bobcat scats at an additional eight loci, within five multiplexes (Table S1). Once again, each locus was amplified a minimum of three times in independent PCRs. All PCRs were conducted using the Qiagen Multiplex Kit (Qiagen, Valencia, CA, USA). Each PCR consisted of a 7 μL reaction containing 1x concentration of Qiagen MasterMix, 0.4 μM of each primer and 0.56 μL of extracted DNA. The only exception was multiplex 2, which contained 0.86 μM of each primer and 1.4 μL of DNA. PCR profiles consisted of a 10 minute 94 °C denaturation step, followed by 30 cycles of 94 °C for 30 s, 55-58 °C for 90 s (Table S1), and 72 °C for 60 s. The exceptions were multiplexes 1 and 4 that were run for 35 cycles. Every reaction concluded with a 60 °C final extension for 10 minutes.

Reding, D.M., Bronikowski, A.M., Johnson, W.E. & Clark, W.R. (2012) Pleistocene and ecological effects on continental-scale genetic differentiation in the bobcat (*Lynx rufus*). *Molecular Ecology,* **21,** 3078-3093.

**Table S1:** PCR multiplexes and the corresponding loci associated with each reaction. PCR profiles and chemistry were consistent across reactions except for the deviations listed in table. Amplification rate is the percentage of PCRs that produced a readable fragment that could be identified as an allele. Genotyping rate is the percentage of scat samples that were successfully genotyped at that locus. The amplification and genotyping rates for multiplexes 3-7 are not reported because they were selectively applied to scats that were successfully amplified at the first two multiplexes. Thus, their error rates are likely biased.

| Multiplex | Locus | Cycles | Annealing temperature (°C) | Primer concentration (μM) | DNA volume (μL) | Amplification rate (%) | Genotyping rate (%) |
| --- | --- | --- | --- | --- | --- | --- | --- |
| 1 | FCA149 | 35 | 55 | 0.4 | 0.56 | 35.3 | 18.0 |
|  | FCA031 |  |  |  |  | 49.3 | 42.0 |
|  | FCA132 |  |  |  |  | 47.4 | 42.0 |
|  |  |  |  |  |  |  |  |
| 2 | FCA043 | 30 | 55 | 0.86 | 0.56 | 41.0 | 38.5 |
|  | FCA082 |  |  |  |  | 44.9 | 40.4 |
|  | LC111 |  |  |  |  | 30.8 | 36.5 |
|  |  |  |  |  |  |  |  |
| 3 | FCA090 | 30 | 55 | 0.4 | 0.56 | N/A | N/A |
|  | FCA096 |  |  |  |  | N/A | N/A |
|  |  |  |  |  |  |  |  |
| 4 | LC109 | 35 | 57 | 0.4 | 0.56 | N/A | N/A |
|  | FCA559 |  |  |  |  | N/A | N/A |
|  |  |  |  |  |  |  |  |
| 5 | BCE5TF | 30 | 58 | 0.4 | 0.56 | N/A | N/A |
|  | FCA740 |  |  |  |  | N/A | N/A |
|  |  |  |  |  |  |  |  |
| 6 | FCA391 | 30 | 57 | 0.4 | 0.56 | N/A | N/A |
|  |  |  |  |  |  |  |  |
| 7 | FCA008 | 30 | 57 | 0.4 | 0.56 | N/A | N/A |
